# Flexural Properties of Renewable Coir Fiber Reinforced Magnesium Phosphate Cement, Considering Fiber Length

**DOI:** 10.3390/ma13173692

**Published:** 2020-08-20

**Authors:** Liwen Zhang, Zuqian Jiang, Hui Wu, Wenhua Zhang, Yushan Lai, Weile Zheng, Jing Li

**Affiliations:** 1 Department of Civil Engineering, Guangzhou University, Guangzhou 510006, China; lwzhang@gzhu.edu.cn (L.Z.); zuqianjiang1996@163.com (Z.J.); whi1223@163.com (H.W.); Ln3maize@163.com (Y.L.); wlzhengaurora@163.com (W.Z.); lj84jch82@163.com (J.L.); 2 Department of Civil Engineering, Nanjing Forestry University, Nanjing 210000, China

**Keywords:** magnesium phosphate cement, flexural toughness, coir fiber, curing age, three-point bending test

## Abstract

Coir fiber (CF), a renewable natural plant fiber, is more competitive in improving poor toughness and crack resistance of magnesium phosphate cement (MPC) than artificial fibers, due to its slight energy consumptions and low costs in production and waste treatment. In this paper, a typical three-point bending test was carried out to study the effects of CF length on MPC flexural properties. A total of forty-two cuboid specimens were employed to investigate the flexural strength, load-deflection behavior, and flexural toughness of MPC, with CF lengths varying from 0 to 30 mm at the curing age of 7 days and 28 days. Results showed that, at both two curing ages, MPC flexural strength first increased with CF length increasing, and then deceased when CF length exceeded the threshold. However, with the increase of CF length, MPC flexural toughness increased continuously, while MPC elastic modulus displayed a decreasing trend. Additionally, Modern micro testing techniques, such as scanning electron microscope (SEM) and X-ray diffraction (XRD), were also used to study the microstructure and phase compositions of specimens for further explaining the themicroscopic mechanism.

## 1. Introduction

Magnesium phosphate cement (MPC), as a new inorganic cementitious material, has great potential in rapidly repairing roads, bridges, and airstrips, due to its advantages of fast setting, high early strength, good durability, and perfect fire resistance [1,2]. However, its drawbacks in toughness and crack resistance severely restrict the application of MPC in practical repair works [3,4]. Existing studies have proven that adding fibers in MPC is a typical and feasible method in improving the drawbacks by reason of fibers’ “bridge effect” and the sharing stress in MPC [4,5,6,7,8]. Nevertheless, most of the fibers currently used in MPC are artificial fibers, such as steel fiber and polymer fiber, which not only are of higher price, but more importantly generate huge energy consumption in their production and waste treatment. Given the severe environmental problems (such as global warming) and high repair work costs introduced by artificial fiber applications, it is very significative to find out a renewable and low-cost fiber to replace artificial fibers.

Reis et al. [9] found that coconut fiber reinforced concrete (CFRC) had greater flexural strength than other natural fiber reinforced concrete. Sekar et al. [10] found that the flexural strength of concrete was obviously improved by adding coir fiber (CF), and the flexural strength increased with curing age increasing. A study conducted by Thanushan et al. [11] demonstrated that CF could greatly improve the residual strength, ductility, flexural toughness, and energy absorption of earth cement blocks. Li et al.’s [12] studies indicated that coir fiber reinforced cementitious composites (CFRCC) possessed higher flexural strength, energy absorbing ability and ductility, and was lighter than conventional cementitious materials. Besides, Rajak et al. [13] hypothesize that randomly oriented coir fiber-reinforced polypropylene composites offer higher mechanical properties than synthetic fiber-reinforced composites. Moreover, as a natural plant fiber, CF has the advantages of better recyclability, biodegradability, and renewability [14,15,16], in addition to a lower price. As a result, CF displays a greater competitiveness in improving MPC flexural properties, compared with artificial fibers. Before applying this natural fiber in practice, it is necessary to clarify the mechanical properties of MPC mingled with CF, by conducting a series of studies aiming at testing CF’s effects on MPC, the authors’ previous study examined the effects of CF volume content on MPC performance. and demonstrated that the CF volume content of 3% is optimal for improving MPC flexural strength and toughness. Then, this study continued to concentrate on examining the effects of CF length on MPC flexural properties. A total of forty-two specimens were employed and divided into two groups, according to the curing age of 7 days and 28 days. A three-point bending test (TPBT) was carried out to investigate the flexural strength, flexural stiffness, flexural load-displacement response, and flexural toughness of CF reinforced MPC, with CF lengths varying from 5 mm to 30 mm. Additionally, modern micro testing techniques of scanning electron microscope (SEM, LEO1530VP, Jena, Germany) and X-ray diffraction (XRD, Inca400, Oxford, UK) were adopted, to study the microstructure and phase compositions of specimens, for further explaining the microscopic mechanism.

## 2. Experimental Program

### 2.1. Raw Material and Mix Design

All specimens had a basic mix proportion employed in the authors’ previous studies [17,18,19], as shown in Table 1. Dead-burned MgO (1500 °C), with a specific surface area of 227.5 m^2^/kg, a density of 2650 kg/m^3^, and a particle size of 45 μm, was produced by Zhengyang Casting Material Company (Xinmi, China), its chemical compositions listed in Table 2. KH_2_PO_4_ and borax from Jiang Hua Chemical Glass Co., Ltd. in Nanjing, China, had been ground for six hours and dried for at least 24 h, before their mixing with MgO. American Society of Testing Materials (ASTM, West Conshohocken, PA, USA) C618 class F FA was employed to replace partial MgO to improve the workability of MPC mortar, with its chemical compositions listed in Table 3.

**Figure 1:**
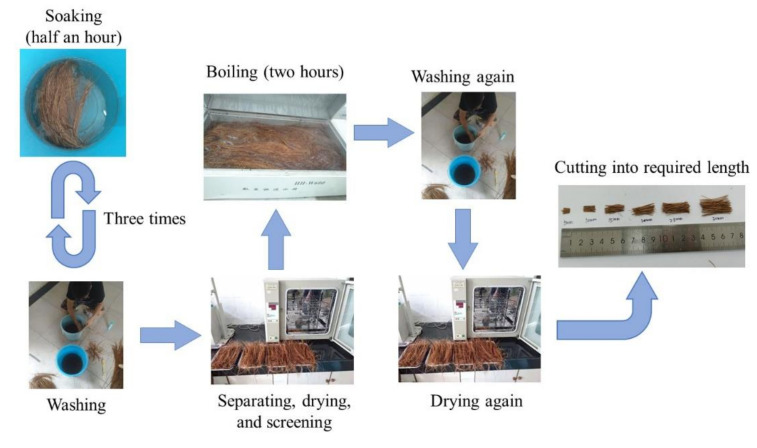
Coir fiber (CF) treating process.

CF from Sri Lanka, provided by Jia Gao Cheng (Import and Export) Trading Co., Ltd. in Shangrao, China, was adopted in this study, its physical properties being presented in Table 4. Before its application, CF should be first soaked in water for 30 min, and then washed for softening itself and removing impurities from its surface. This soak-wash step was repeated at least 3 times, until CF was cleaned and intenerated completely. Then, CF was separated from each other by a steel comb and dried in a drying oven for 10–12 h at thirty degrees celsius. The CF, with a relatively uniform diameter along its axis, was extracted and suffered a boil-wash process until water ran clear. At last, these fibers were dried again, and then cut to the lengths of 5 mm, 10 mm, 15 mm, 20 mm, 25 mm, and 30 mm, respectively. Figure 1 displays the whole treating process of CF.

### 2.2. Test Specimens

**Figure 2:**
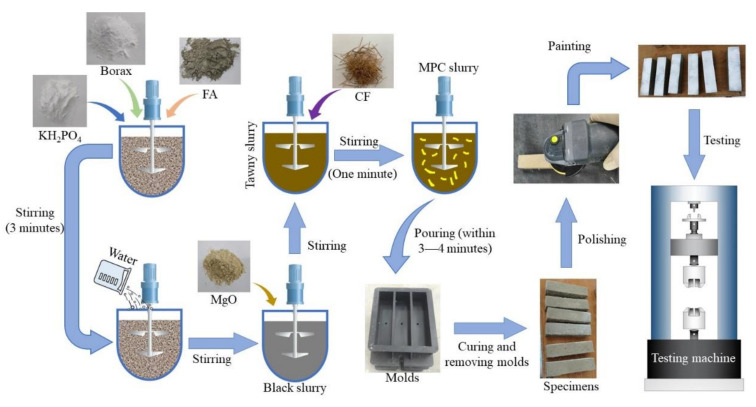
Specimen preparation.

The specimens’ preparation process was as follows: potassium dihydrogen phosphate (KH_2_PO_4_), borax, and fly ash (FA) were first mixed before water was poured in. Then, magnesium oxide (MgO) was added into the homogeneous black slurry, comprising water and the mixture. When the slurry turned from black to tawny, CF was dusted into the slurry and stirred for about 1 min. Finally, the mixed MPC slurry was pumped into the molds within 3–4 min. When the MPC slurry was solidified completely (after approximately 30–60 min), specimens were demolded and then cured in the time corresponding to the test requirement. Figure 2 explains the whole preparation process mentioned above.

**Figure 3:**
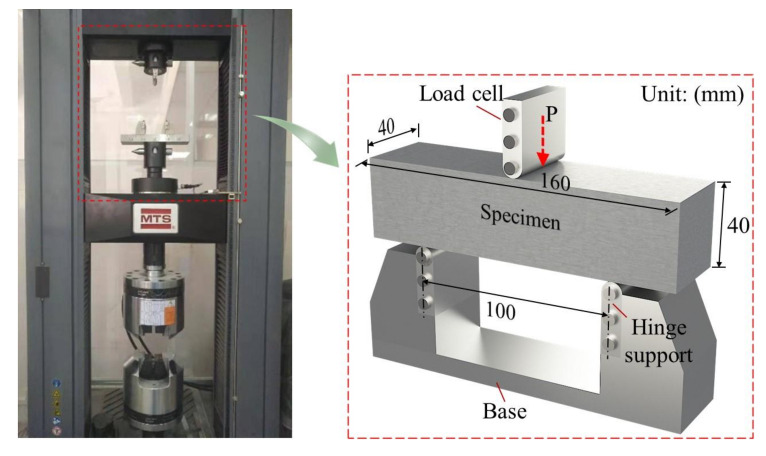
Specimen and test setup.

A total of forty-two rectangular MPC specimens with the dimension of 40 mm × 160 mm × 40 mm, were employed in TPBT, referring to GB/T 17671-1999 [20], as shown in Figure 3. These forty-two specimens were divided into two groups respectively, according to the two curing ages of 7 days and 28 days. The twenty-one specimens in each group were divided into seven sets according to different CF lengths (0 mm, 5 mm, 10 mm, 15 mm, 20 mm, and 25 mm). Each set comprised three identical specimens, which were named following the order of CF length-curing age-specimen serial number in this set. E.g., CFL5-T7-1 means the first specimen of the set with a CF length of 5 mm at the curing age of 7 days. Basic proportion of CF in each specimen was defined as 3% (volume concentration), based on the authors’ previous study. More details of specimens are listed in Table 5, in which L and VC mean the length and volume concentration of CF, respectively. Additionally, the sets of CFL0-T7 and CFL0-T28 were specimens without CF, and were thus specified as reference specimens.

### 2.3. Test Method

Specimens were tested with a universal testing machine (MTS-E45.305, Shenzhen, China) shown in Figure 3, following ASTM-C1609 [21], and were polished and then painted by white color (Figure 2) for ease of capturing their failure processes. Each specimen was installed on a steel base with two hinge supports with a center distance of 100 mm, and each support 30 mm from the edge of the specimen near it, as described in Figure 3.

A specimen was preloaded firstly at a rate of 2.0 mm/min according to the loading cell displacement, until loading force increased to 0.01 kN, when the load cell, the specimen, and the base had been center aligned, as shown in Figure 3. Then, the specimen was loaded at a rate of 1.0 mm/min, during which time the load-deflection response at the midspan was recorded by the sensor installed in the load cell. Meanwhile, its fracture process was recorded by a high-definition camera set in front of it. The test would be terminated when the loading force declined to ten percent of its peak value.

After that, sample pieces were taken from failed specimens and tested using SEM and XRD. Thus, the microstructure and phase constitution of MPC were characterized for understanding the changing mechanism of MPC performance with CF length.

## 3. Results and Discussions

### 3.1. Failure Process and Modes

**Figure 4:**
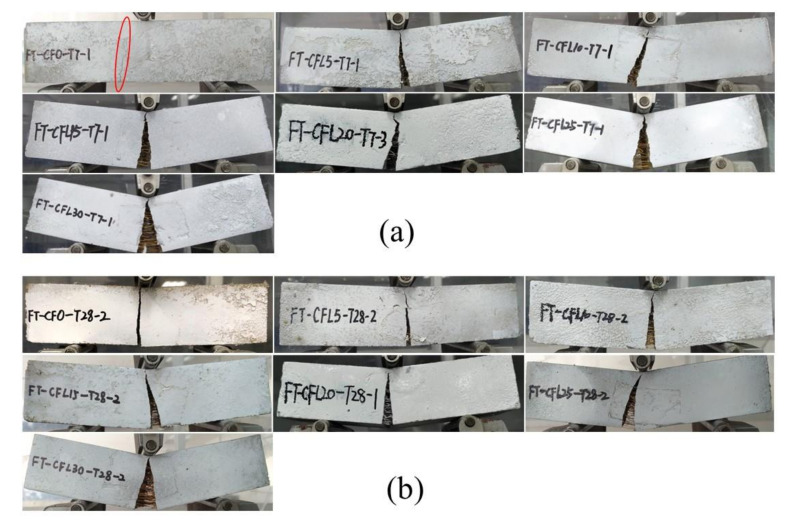
Failure modes: (**a**) T7; (**b**) T28.

Figure 4a,b provide the failure mode of specimens at two curing ages, respectively. It should be noted that one typical specimen was selected from each set to represent the failure mode of the set.

**Figure 5:**
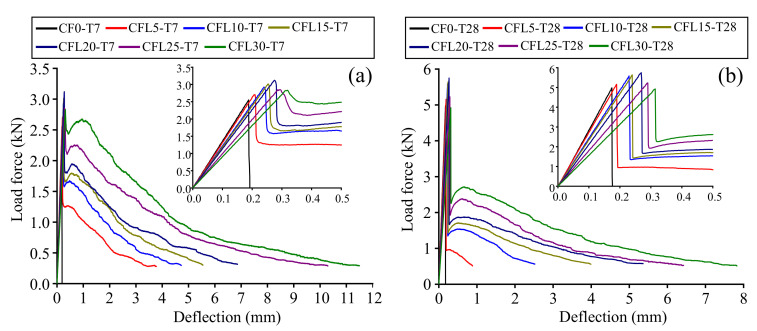
L-D curves: (**a**) T7; (**b**) T28.

For group T7, specimens presented a typical brittle failure when they were not mixed with CF (set CF0-T7). Before the specimens failed, no obvious failure signs could be noticed, except for a fissure, which occurred at the midspan of the specimens’ bottom shortly after loading. Then, the fissure expanded fleetly through the specimens and broke them in seconds with a rock-fissuring sound. Few pieces fell off from the specimens. However, some symptoms of ductile failure appeared when CF was added into specimens, with a tendency to be more distinct accompanying the increase of CF length. For example, in the sets of CFL5-T7--CFL15-T7 in Figure 4a, a crack occurred gradually and grew slowly as CF length increased, with soft CF-rupturing and MPC-cracking sounds heard sometimes. Correspondingly, the crack propagated to a wider cleft, separating the specimens into two parts, which were nevertheless still connected by a few CF in the clefts. In addition, the deflection of the specimens was larger than that of CF0-T7, and increased with CF length growing, which could also be found in the load-deflection (L-D) curves of specimens (Figure 5a). When CF length exceeded 20 mm, i.e., in set CFL20-T7, CFL25-T7, and CFL30-T7, specimens exhibited an obvious typical ductile failure. The whole failure process was tardy, with a crack crawling from the specimens’ bottom to the top at midspan, with a significant deflection, and eventually developing to a wider interstice. Frequent fracture sounds of CF and MPC were heard clearly in turn. Most CF still stayed in the interstice to prevent specimens from breaking down and MPC pieces from peeling off from the specimens, as in Figure 4a.

**Figure 6:**
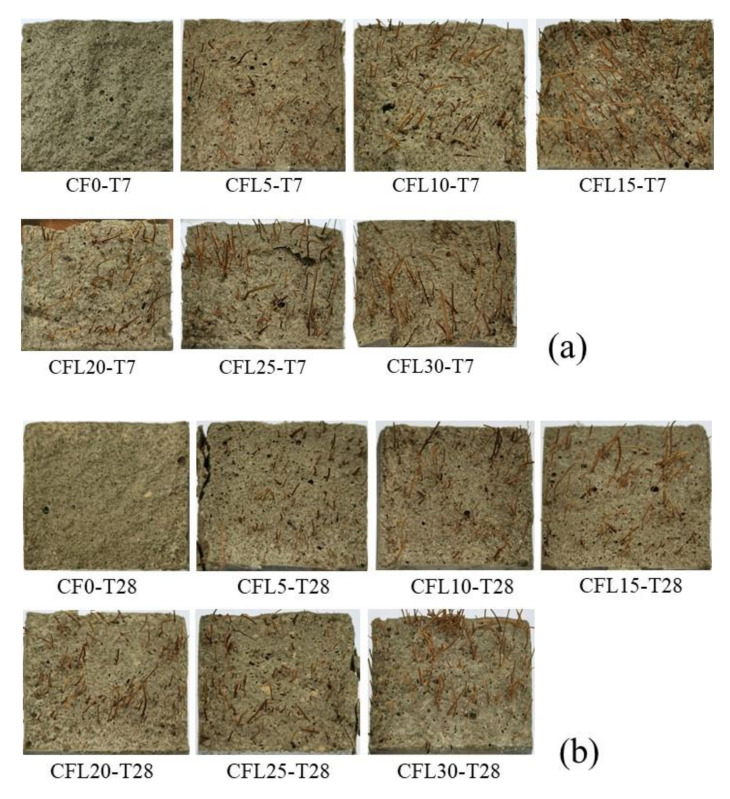
Section of failed specimens: (**a**) T7; (**b**) T28.

T28 presented a similar effect of CF on failure modes as T7, i.e., the modes shifted from typical brittleness to typical ductility, as shown in Figure 4b. However, T28 was more brittle than T7 for specimens with the same CF length. Each specimen comprising CF in T28 displayed a smaller deflection and thinner cracks than the corresponding specimen in T7. This larger brittleness of T28 compared with T7 could also be observed in the L-D curve of specimens. As in Figure 5b, for specimens mixed with CF, the L-D curve of T28 had a sharper softening branch than that of T7 with the same CF length. This increased brittleness benefited from the fuller hydration of MPC under long curing age, which resulted in a compacter internal structure for specimens. Figure 6a,b offer the section of failed specimens at 7 days and 28 days, respectively, which clearly shows that T28 had a less porous fracture surface than T7. However, although capable of improving the loading capacity of specimens this compacter was, it lowered their ductility yet. In addition, as in Figure 6, most CF were pulled apart in T28, due to a higher CF-MPC bonding strength caused by the fuller hydration, releasing larger energy per unit time, compared with the gradually pulled-out CF from specimens in T7, and leading to T28 behaving more brittlely than T7 as well.

### 3.2. L-D Curves

**Figure 7:**
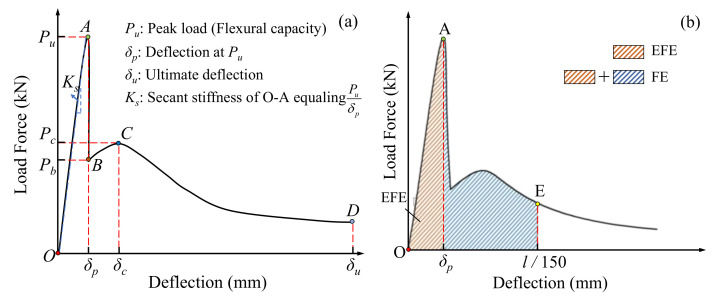
Schematic of load-deflection (L-D) behavior: (**a**) L-D curve configuration; (**b**) Flexural toughness calculation.

Figure 7a shows a curve comprising four branches to represent the configuration of all specimen L-D curves, namely the approximately straight ascending branch (O--A), the sharp descending branch (A--B), the secondary curvilinear ascending branch (B--C), and the secondary gradual descending branch (C--D), respectively. For specimens without CF, their L-D curves only had the branches of O--A and A--B, corresponding to the typical brittleness observed from the specimens in the test, as illustrated in Section 3.1. For specimens with CF, they presented a complete L-D curve, consisting of the four branches, which had a respectively similar trend, with CF length increasing at two curing ages.

**Figure 8:**
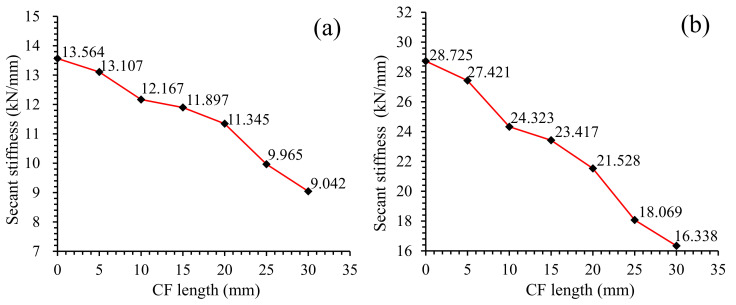
Secant stiffness of L-D curve: (**a**) T7; (**b**) T28.

In branch O--A--B, as in Figure 5, loading forces of all specimens grew up to the peak load (point A) in approximately a linear way, and then dropped sharply to point B. The slope of branch O--A, i.e., the secant stiffness *K_s_* of specimens shown in Figure 8, decreased with CF length increasing. Take group T28 as an example—Ks declined from 28.8 kN/mm to 16.3 kN/mm as CF length increased from 0 mm to 30 mm. Compared with Ks, the peak load of specimens displayed a trend of rising first and then falling. When CF length increased to 20 mm, the peak load grew to its maximum value (3.12 kN and 5.75 kN for T7 and T28, respectively), and then decreased in turn, after CF length exceeded 20 mm. This phenomenon indicates that the ability of CF in improving MPC capacity was restricted by its length. A detailed explanation is provided in Section 3.3 and Section 3.5.

When curves dropped to point B, as in Figure 5, they would decline continuously for specimens without CF (CF0-T7 and CF0-T8), due to the rapid expansion of cracks in specimens. However, for specimens with CF, curves ascended again from point B to C, resulting from the added CF restricting the cracks through its “bridge effect”, described in Section 3.3. Moreover, the force at point B, the force at point C, and the force increment between these two points all increased with CF length growing. For example, as in Figure 5a, the force at point C increased from 1.26 kN to 2.67 kN, as CF length grew from 5 mm to 30 mm, which could be attributed to the increasing bond force between CF and MPC due to a greater surface area of CF brought by CF length growing. This increasing bond force naturally enhanced the “bridge effect”, stopping the degradation of MPC capacity earlier, and allowing the specimens to have larger secondary capacity.

When loading force increased to point C, however, the tension of CF exceeded its tensile strength or the bonding force, leading to the rupture or pulling out of CF. As a result, specimen capacity declined again, until the specimen was broken (point D). Additionally, branch C--D declined more gradually in the case of longer CF. For example, as in Figure 5a, the distance of branch C--D increased from 3.37 mm to 10.57 mm, when CF length grew from 5 mm to 30 mm, implying that longer CF contributes to improving the ductility of specimens, which could also be verified by the slower failing process of the specimens, and the frequent rupturing noises of CF observed in the test.

Although CF length had similar effects on the L-D behavior of specimens at two curing ages, it had a lower effectiveness in improving specimen ductility at longer curing age. As in Figure 5, T28 exhibited slower softening branches (C--D), larger deflections and larger secant stiffness than T7 with the same CF length, which were resulted from the compacter structure of MPC caused by the fuller hydration at longer curing age. Because this compacter structure could improve the bonding strength between CF and MPC besides the elastic modulus of MPC itself, most CF was ruptured rather than pulled out for T28, as in Figure 6, to release more energy per unit time, resulting in lower ductility for T28 than T7 with the same CF length.

### 3.3. Flexural Strength

**Figure 9:**
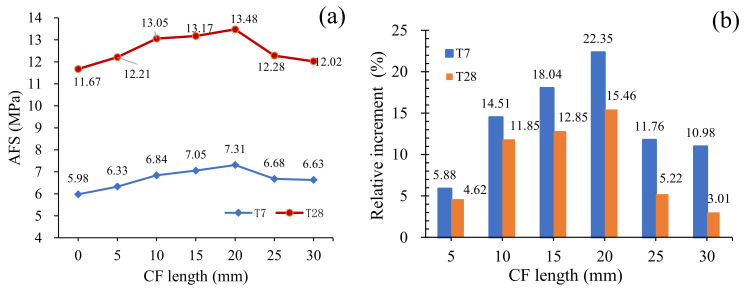
Average flexural strength: (**a**) AFS; (**b**) Relative increment of AFS.

Figure 9 offers the average flexural strength (AFS) of specimens, which was calculated by Equation (1), following ASTM C293 [22].

(1) $$f_{cl}=\frac{3P_{u}L}{2bd^{2}}$$

where *f_cl_* is the flexural strength, *P* is the peak load, *L* is the support distance (100 mm), *b* is the specimen width (40 mm), and *d* is the specimen depth (40 mm). As well as the stress-strain behavior, AFS was improved after adding CF and increased with CF length increasing for all specimens at both curing ages. However, this increase of AFS was cut off when CF length grew to a certain value, and then declined in turn as CF length increased continuously. For example, As in Figure 8a,b, AFS of T28 increased from 11.67 MPa to 13.48 MPa, and grew by 15.46% when CF length raised from 0 mm to 20 mm, but decreased from 13.48 MPa to 12.02 MPa, as CF length kept on rising to 30 mm.

**Figure 10:**
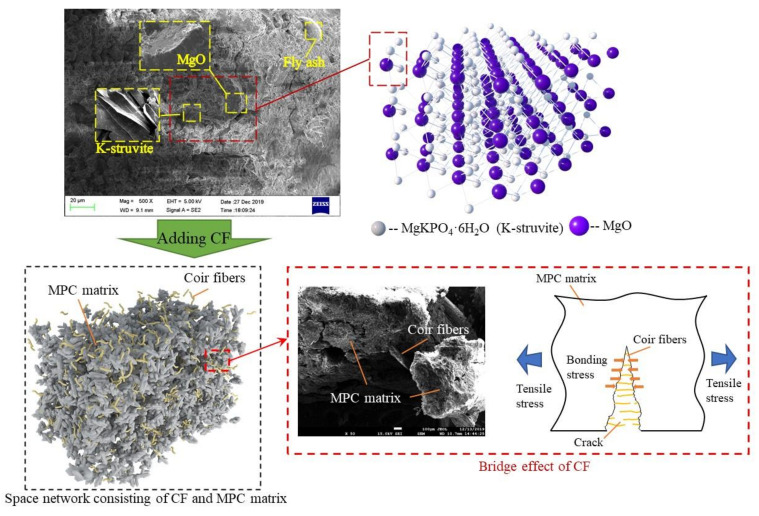
Micro-structure of magnesium phosphate cement (MPC) matrix and schematic of bridge effect.

These phenomena could be ascribed to the following reasons. In fact, MPC matrix, as a space network assembled by hydration crystals comprising unreacted MgO particles and a compound named “potassium phosphate magnesium (MgKPO_4_·6H_2_O, MKP)” (also known as K-struvite) around them, as shown in Figure 10, has a greater capacity of resisting compression, but a lower possibility of resisting to tension, due to the worse performance of MKP-MgO interface and MKP itself under tensile stress, just like concrete and cement mortar, a having lower tension capacity because of calcium silicate hydrate (CSH)’s poor tensile properties. However, after adding CF, the perfect bonding performance between CF and MKP together with larger CF tensile strength can ensure the strong connection of the hydration crystals by CF, allowing CF to withstand the tensile stress supposed to be in MKP. Figure 10 presents the schematic of the “bridge effect” mechanism mentioned in Section 3.1 and Section 3.2, which would be enhanced by improved bonding force between CF and MKP as CF length grew. As a result, AFS also increases with the increase of CF length. However, longer CF leads to the decline of its amount in the matrix of per volume, due to its fixed proportion, thereby reducing its effectiveness in improving MPC flexural strength. Given that overlong CF may twine and agglomerate together, thereby resulting in its nonuniform distribution and the lower fluidity of MPC mortar, specimens would subsequently have larger local pores and a looser structure, as shown in Figure 5. In addition to the effect of CF length on specimen structure, the increase of CF length suppresses the production of MKP, as described in Section 3.5. So, because of all the effects mentioned above on the mechanical properties of specimens, the flexural strength consequently decreased in turn after CF length exceeding 20 mm.

Although curing ages had little effect on the specimen’s flexural strength with CF length increasing, a longer curing age would yet result in a larger flexural strength for the same CF length, due to the greater bonding performance between CF and MPC with the help of MPC fuller hydration. Additionally, the relative increments of flexural strength for group T28 was smaller than that of group T7, which could be proven by the cases of 20 mm long CF, as in Figure 9b, where AFS increased by 15.46% in CFL20-T28, but improved by 22.35% in CFL20-T7, due to the larger flexural strength of CF0-T28 than CF0-T7.

### 3.4. Flexural Toughness

**Figure 11:**
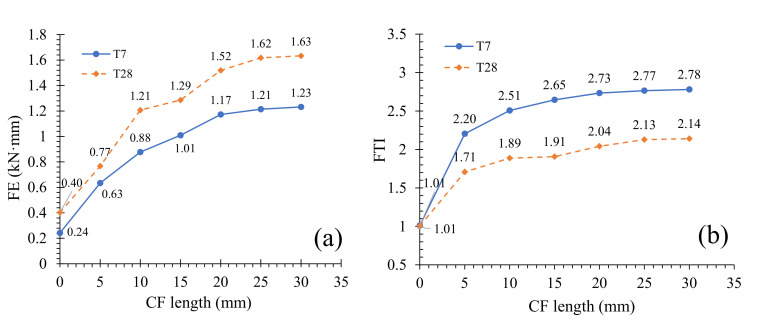
Flexural toughness: (**a**) flexural energy absorption (FE); (**b**) effective flexural toughness (FTI).

To evaluate the effect of CF length on MPC flexural properties, Figure 11 offers the flexural toughness of specimens, calculated by the area of branch O--A--E shown in Figure 6b, in reference to ASTM C1609 [21] and the study by Chen et al. [23], which represented the total flexural energy absorption (FE) of a specimen in the test. Additionally, an index named the “effective flexural toughness” (FTI) was defined in order to understand CF effects on the energy absorption of specimens in different stress-strain branches according to Equation (2),

(2) $$\mathrm{FTI}=\frac{\mathrm{FE}}{\mathrm{EFE}}$$

where the abbreviation “EFE” is the effective flexural energy absorption, representing the energy absorption in the ascending branch O--A.

As in Figure 11a, FE grew as CF length increased at two curing ages, demonstrating that the flexural toughness of specimens is improved. However, this improvement decreased when CF length was larger than a value. It could be observed clearly from Figure 11a that there existed a turning point in the FE curve, which moved backward with curing age increasing. For T7, FE increased from 0.24 J to 1.17 J before the turning point of 20 mm, with an average relative increasing rate (ARIR) of 19.375% per meter CF. When CF length increased after the turning point from 20 mm to 30 mm, FE increased from 1.17 J to 1.23 J with an ARIR of 0.52% per meter CF. But for T28, the turning point was postponed to 25 mm. In this case, FE increased from 0.4 J to 1.61 J as CF length grew from 0 to 25 mm, with an ARIR of 15.1% per meter CF; and increased from 1.61 J to 1.63 J as CF length grew from 25 mm to 30 mm, with an ARIR of 0.29% per meter CF. These demonstrate that CF length is more effective in improving the flexural toughness of specimens at longer curing age.

FTI ascended simultaneously with CF length growing, as Figure 11b shows. Taking T28 for example, FTI increased to 2.14 and by 111.9% relative to the value of specimens without CF, when CF length grew to 30 mm. In fact, the increase of FTI indicates the limited effectiveness of CF length on the ascending branch O—A O--A, due to tiny cracks in the matrix, as described in Section 3.2. However, the extra effectiveness of CF length was spent in improving the softening branch of specimens. The increase of FTI not only suggests that the softening branch will absorb more energy with CF length increasing, but also demonstrates the improvement of specimen ductility. These conclusions are consistent with the suggestions expressed by stress-strain behaviors illustrated in Section 3.2.

### 3.5. Microstructure Analysis

**Figure 12:**
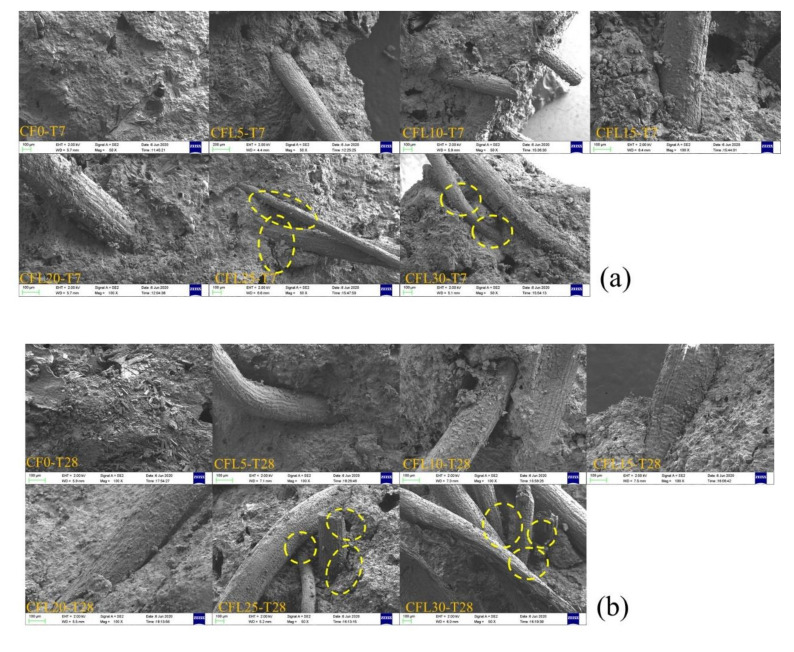
Scanning electron microscope (SEM) of specimens: (**a**) T7; (**b**) T28.

SEM and XRD analyses were carried out in order to investigate the effect of CF length on MPC micro-structure and hydration. Figure 12a,b offer the SEM result of specimens at two curing ages, respectively, which show a similar trend of micro-structure for T7 and T28. In the case of CF shorter than 20 mm, no significant change was observed in the micro-structure of specimens with CF length increasing, except for gradually increased micro-porosity, which led to a lower compactness as described in Section 3.1 (Figure 4). However, for overlong CF, e.g., 25 mm and 30 mm, they twined and agglomerated together, as marked by the dotted yellow line in Figure 11, resulting in a loose structure around the MPC-CF interface and larger local pores.

**Figure 13:**
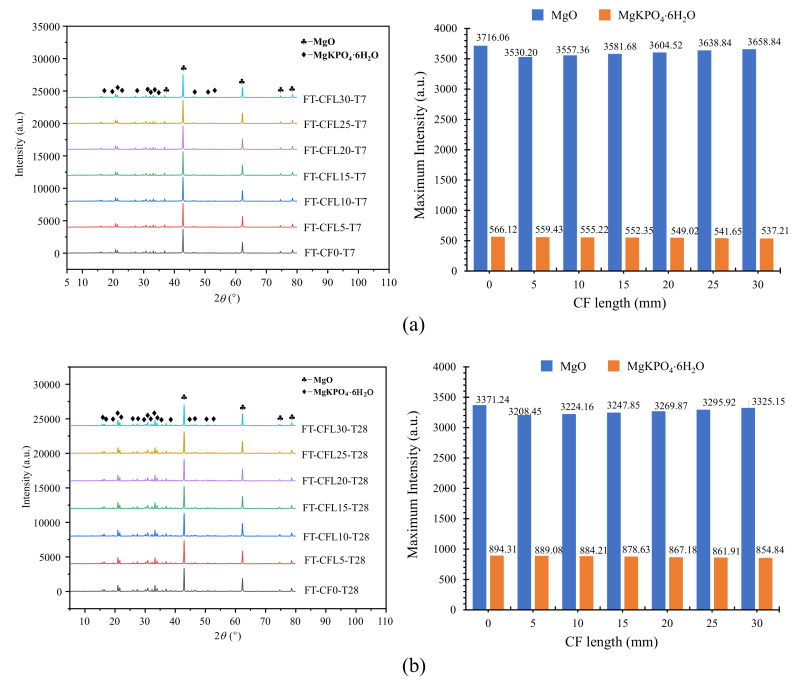
X-ray diffraction (XRD) of specimens: (**a**) T7; (**b**) T28.

**Figure 14:**
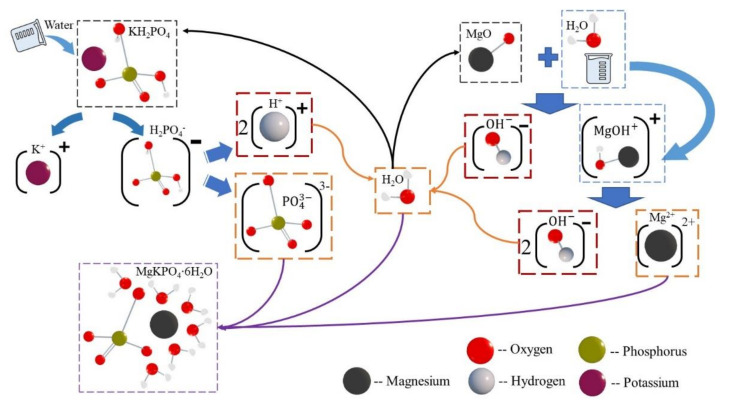
Hydration process of MPC.

Moreover, the XRD result in Figure 13 showed that the amount of MKP decreased with CF length increasing. For T7, the maximum intensity of MKP decreased from 566.12 a.u. to 537.21 a.u., as CF length grew from 0 mm to 30 mm; for T28, the maximum intensity dropped from 894.31 a.u. to 854.84 a.u. This phenomenon could be explained by the hydration process of MPC [24,25]. In this process, KH_2_PO_4_ is dissolved into the potassium ion (K^+^) and the dihydrogen phosphate ion (H_2_PO_4_^−^) first, as in Figure 14. Then, H_2_PO_4_^−^ is further dissolved into the hydrogen ion (H^+^) and the phosphate ion (PO_4_^3−^). Meanwhile, MgO is converted into the hydroxyl ion (OH^−^) and the magnesium ion (Mg^2+^) by the chemical reaction with water. This Mg^2+^ finally combines K^+^, PO_4_^3−^, and the bound water coming from the reaction of H^+^ and OH^−^, to produce the hydration product MKP. Additionally, the excess bound water continues to engage in the hydrolysis reaction of KH_2_PO_4_ and MgO together with poured water. However, this cycle hydration process will be disturbed by adding CF. CF will absorb lots of water in the hydration reaction, slowing down the hydration. Furthermore, the longer CF is, the more water is absorbed, due to the increased CF surface area. As a result, MgO and KH_2_PO_4_ cannot obtain sufficient water to keep the reaction going on to produce more MKP with CF length increasing, which can also be demonstrated by the growing MgO shown in Figure 13.

From the analysis for SEM and XRD described above, it can be known that longer CF will lower the compactness of MPC and reduce the amount of MKP, although it contributes to a stronger connection between the MPC matrix. Consequently, the improvement of MPC flexural strength and toughness caused by adding CF will decline when CF is longer than a threshold (20 mm in this study), as reported in Section 3.1, Section 3.2, Section 3.3 and Section 3.4.

## 4. Conclusions

A typical three-point bonding test was employed in this study to investigate the effect of CF length on the flexural performances of MPC, including flexural strength, loading-deflection behaviors, and flexural toughness. Additionally, the microstructure analysis by means of SEM and XRD were conducted for further explaining the microscopic mechanism. Conclusions are summarized as follows:

(1) CF length presents similar effects on the flexural performances of MPC at different curing ages. However, specimens at the curing age of 28 days exhibit higher flexural strength, stiffness, and flexural toughness than specimens at the curing age of 7 days.
(2) The flexural strength of MPC can be improved by increasing the CF length within a certain length range, but begins to decrease when CF is longer than 20 mm (in this study). However, further tests are needed to capture a more precise threshold of CF length.
(3) Longer CF is more beneficial to the improvement of MPC ductility, but reduces MPC stiffness continuously. In addition, MPC performance displays a secondary rise after its softening first as CF length increases.
(4) CF contributes to improving MPC toughness. However, this improvement slows down after CF is longer than a threshold. In this study, the threshold is 20 mm for T7 and 25 mm for T28. Moreover, overlong CF has slight extra help in strengthening MPC’s crack-resistance in its elastic stage than CF with the threshold length.
(5) Longer CF reduces the amount of MKP and degrades the compactness of MPC matrix.
(6) The threshold of CF length suggested in this study may change with the scaling of specimens, which will be investigated in further tests.

## Figures and Tables

**Table 1:** Mix proportion of magnesium phosphate cement (MPC) (kg/m^3^).

MgO	KH_2_PO_4_	Borax	FA	Water
1171.87	797.26	117.18	295.31	328.3

**Table 2:** Chemical compositions of MgO.

**Compositions**	MgO	Al_2_O_3_	Fe_2_O_3_	CaO	SiO_2_	LOI
**Weight Percent (%)**	96.25	0.29	1.09	1.18	1.16	0.03

**Table 3:** Chemical compositions of fly ash (FA).

**Compositions**	SiO_2_	Al_2_O_3_	Fe_2_O_3_	CaO	TiO_2_	MgO	SO_3_	LOI
**Weight Percent (%)**	56.74	24.58	6.55	4.87	1.86	3.3	0.8	1.3

**Table 4:** Physical properties of coir fiber (CF).

Diameter (µm)	Density (kg/m^3^)	Tensile Strength (MPa)	Elasticity Modulus (GPa)	Elongation (%)
150–350	1200	112–146	2.3–3.4	14–28

**Table 5:** Specimen details.

Group	Set	Specimen Number	Curing Age (Day)	CF		
				*L*^1^ (mm)	*VC*^2^ (%)	Mass (g)
T7	CF0-T7	FT-CF0-T7-1	7	0	0	0
		FT-CF0-T7-2				
		FT-CF0-T7-3				
	CF5-T7	FT-CFL5-T7-1		5	3	9
		FT-CFL5-T7-2				
		FT-CFL5-T7-3				
	CF10-T7	FT-CFL10-T7-1		10		
		FT-CFL10-T7-2				
		FT-CFL10-T7-3				
	CF15-T7	FT-CFL15-T7-1		15		
		FT-CFL15-T7-2				
		FT-CFL15-T7-3				
	CF20-T7	FT-CFL20-T7-1		20		
		FT-CFL20-T7-2				
		FT-CFL20-T7-3				
	CF25-T7	FT-CFL25-T7-1		25		
		FT-CFL25-T7-2				
		FT-CFL25-T7-3				
	CF30-T7	FT-CFL30-T7-1		30		
		FT-CFL30-T7-2				
		FT-CFL30-T7-3				
T28	CF0-T28	FT-CF0-T28-1	28	0	0	0
		FT-CF0-T28-2				
		FT-CF0-T28-3				
	CF5-T28	FT-CFL5-T28-1		5	3	9
		FT-CFL5-T28-2				
		FT-CFL5-T28-3				
	CF10-T28	FT-CFL10-T28-1		10		
		FT-CFL10-T28-2				
		FT-CFL10-T28-3				
	CF15-T28	FT-CFL15-T28-1		15		
		FT-CFL15-T28-2				
		FT-CFL15-T28-3				
	CF20-T28	FT-CFL20-T28-1		20		
		FT-CFL20-T28-2				
		FT-CFL20-T28-3				
	CF25-T28	FT-CFL25-T28-1		25		
		FT-CFL25-T28-2				
		FT-CFL25-T28-3				
	CF30-T28	FT-CFL30-T28-1		30		
		FT-CFL30-T28-2				
		FT-CFL30-T28-3				

^1, 2^*L* means the length of CF; *VC* represents the volume concentration of CF.
